# Exploring the significance of extracellular vesicles: Key players in advancing cancer and possible theranostic tools

**DOI:** 10.1016/j.cpt.2024.04.005

**Published:** 2024-04-25

**Authors:** Bhaumik Patel, Shreyas Gaikwad, Sahdeo Prasad

**Affiliations:** a Department of Immunotherapeutic and Biotechnology, Texas Tech University Health Science Center, Abilene, TX 79601, USA; b Masonic Cancer Center, University of Minnesota, Minneapolis, MN 55455, USA

**Keywords:** Extracellular vesicle (EV), Cancer metastasis, Biomarker, Bioengineered EV

## Abstract

Metastasis remains a critical challenge in cancer treatment and the leading cause of cancer-related mortality. Ongoing research has demonstrated the key role of extracellular vesicles (EVs) in facilitating communication between distant organs. Cancer cells release a substantial number of EVs that carry distinct cargo molecules, including oncogenic proteins, DNA fragments, and various RNA species. Upon uptake, these cargo molecules profoundly influence the biology of both normal and cancerous cells. This review consolidates the understanding of how EVs promote tumorigenesis by regulating processes such as proliferation, migration, metastasis, angiogenesis, stemness, and immunity. The exploration of EVs as a non-invasive method for cancer detection holds great promise, given that different cancer types exhibit unique protein and RNA signatures that can serve as valuable biomarkers for early diagnosis. Furthermore, growing interest exists in the potential bioengineering EVs for use as prospective therapeutic tools for cancer treatment.

## Highlights

• Extracellular vesicles (EVs) play pivotal roles in cancer from inception through all phases of development.
• EV-mediated signaling profoundly influences the tumor microenvironment (TME), fostering immune evasion.
• EV-based liquid biopsies are promising when biopsy sampling is challenging.
• Engineered EVs show potential for targeting tumors more precisely.

## Graphical abstract

**Figure:**
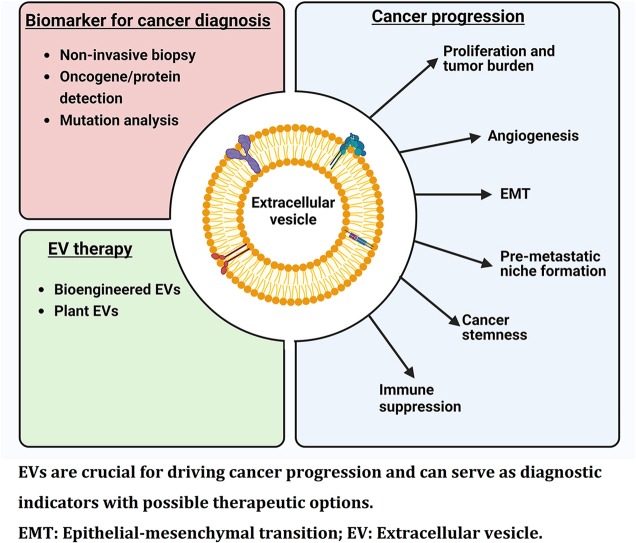

## Introduction

**Figure 1:**
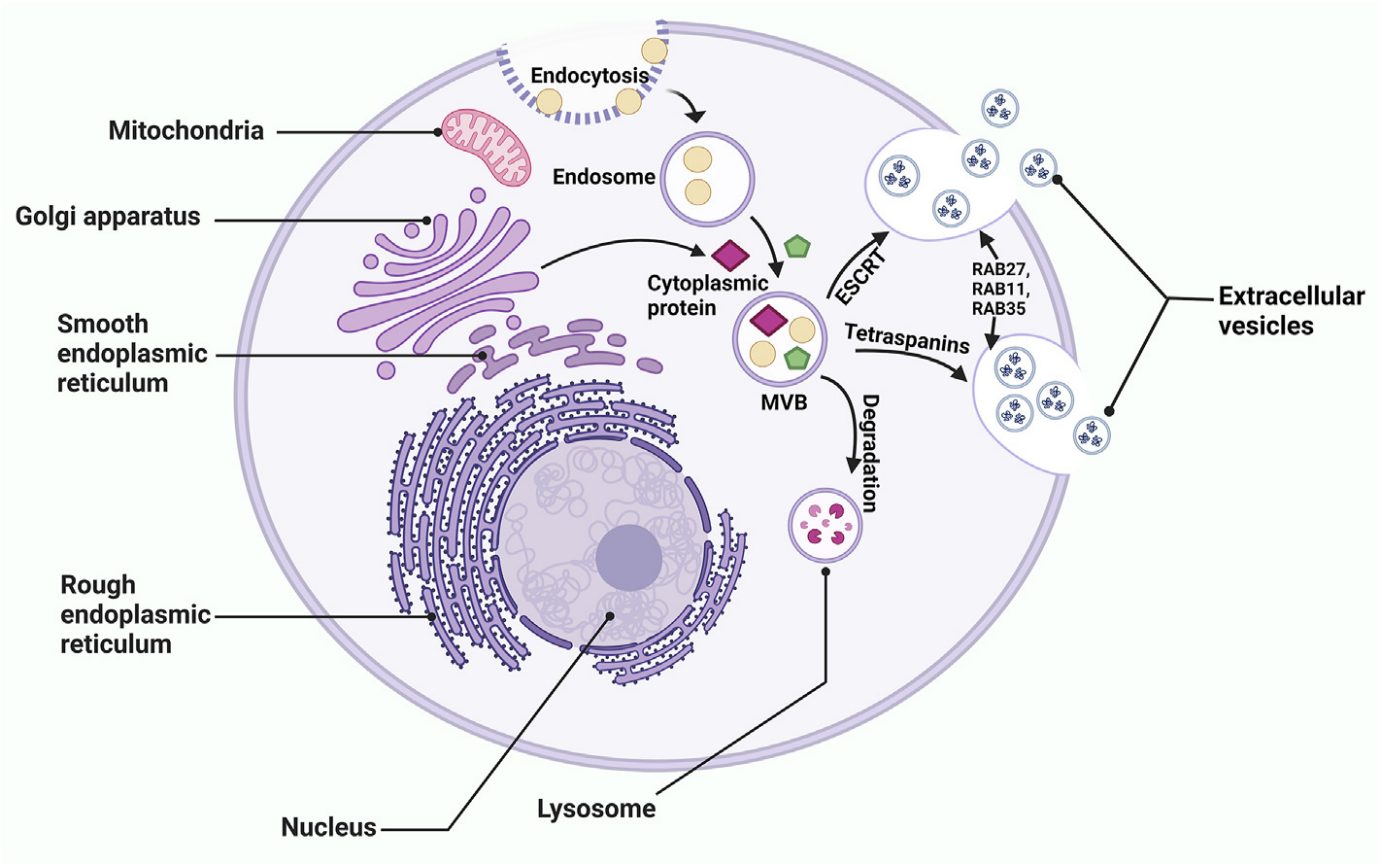
EV biogenesis and release. EV formation mainly depends on ESCRT-dependent and ESCRT-independent pathways. Endosomes form MVBs upon endocytosis. MVBs can be processed by ESCRT or tetraspanins, and RAB27, RAB11, and RAB35 can direct MVBs toward the plasma membrane. MVBs fuse with the plasma membrane and are released as EVs. ESCRT: Endosomal sorting complex required for the transport; EV: Extracellular vesicle; MVB: Multivesicular body; RAB: Member of the RAS oncogene family.

Cell-to-cell communication is an established phenomenon, and multiple extracellular communication pathways have been demonstrated. The roles of extracellular vesicles (EVs) in distant cell communications have been widely studied over recent decades. Numerous findings have demonstrated the critical roles of EVs in transferring biomolecules to distant organs. EVs form a heterogeneous group of lipid-bound, cell-derived membranous structures comprising exosomes and microvesicles. EV formation originates with the endosomal pathway, where endosomes are trafficked and processed by the endosomal sorting complex required for transport (ESCRT) machinery, member of the RAS oncogene family (RAB)7 and tetraspanin proteins to generate multivesicular bodies (MVBs) containing intraluminal vesicles (ILVs). MVBs are then directed to the plasma membrane by RAB27, RAB11, or RAB35, where they fuse with the plasma membrane and are released as EVs [Figure 1]. EVs carry various components including lipids, proteins, DNA fragments, and different RNA species (coding and non-coding RNAs). These EVs are known to carry a plethora of biomolecules, including messenger RNAs (mRNAs), microRNAs (miRNAs), long noncoding RNAs (lncRNAs), and small-interfering RNAs (siRNAs).^1^

Cancer stem cells (CSCs) secrete large amounts of EVs with diameters ranging from 40 to 150 nm. These EVs function in a unique form of intercellular communication that can promote cell growth and survival, increase cancer stemness, increase invasive and metastatic activities, and regulate immune responses and the premetastatic niche. These unique properties make EVs excellent tools for studying the roles of CSCs cancer progression in detail. Thus, we reviewed the importance of EVs in cancer development. In the future, bioengineered EVs could be used to treat multiple diseases, given that EVs are biological components of bodily fluids that can cross all biological barriers.

## Extracellular vesicles regulate cancer stem cell initiation and expansion

CSCs, originally known as tumor-initiating cells, are derived from stem cells that have undergone a malignant transformation and can interact with surrounding stromal cells via EVs. During the early stages, CSCs are surrounded by heterogeneous cells. These CSCs secrete EVs containing miRNAs associated with the RNA-induced silencing complex (RISC)-loading complex (RLC), as well as exosomal shuttle RNA (esRNA), which can potentially convert non-tumorigenic cells into tumor-forming cells. Patel et al.^2^ demonstrated that *Tuberous Sclerosis 1* (*TSC1*)-null cells delivered esRNAs against neurogenic locus notch homolog protein 1 (Notch1) and a Ras homolog enriched in the brain (Rheb) to neighboring wild-type cells and rendered them functionally similar to *TSC1*-null cells. These findings have significant clinical implications because a relatively small population of mutated CSCs can affect numerous neighboring cells with an intact genome. Thus, genetic alterations directly affecting single cells may influence the functions of all surrounding cells and the organ system, leading to the acceleration of several disease processes.^2^

Moreover, Melo et al.^3^ demonstrated that cancer EVs contain pre-miRNA and RLC proteins such as Dicer, TRBP, and AGO2. Upon the transfer of these EVs into non-tumorigenic epithelial cells, RLC proteins such as Dicer convert pre-miRNA to mature miRNA, thereby altering the transcriptome of target cells in a Dicer-dependent manner. In this manner, EVs from CSCs can initiate tumor initiation.^3^

## The functions of extracellular vesicles in cancer progression

Ongoing advances in cancer biology, coupled with new approaches for exploring cancer progression, have uncovered key molecular mechanisms whereby EVs play pivotal roles. A comprehensive understanding of cancer progression and metastasis involves various stages such as cancer cell proliferation, local tissue invasion, intravasation (migration), survival in the circulation (involving the epithelial–mesenchymal transition [EMT] and cancer cell stemness), premetastatic niche formation, and colonization. The involvement of EVs in multiple steps of cancer progression has been well documented. A more profound understanding of how EVs modulate carcinogenesis holds important potential for advancing cancer management and developing novel therapeutics. Therefore, we elucidated the interplay between EVs and cancer progression.

### Cancer cell proliferation driven by extracellular vesicles

**Figure 2:**
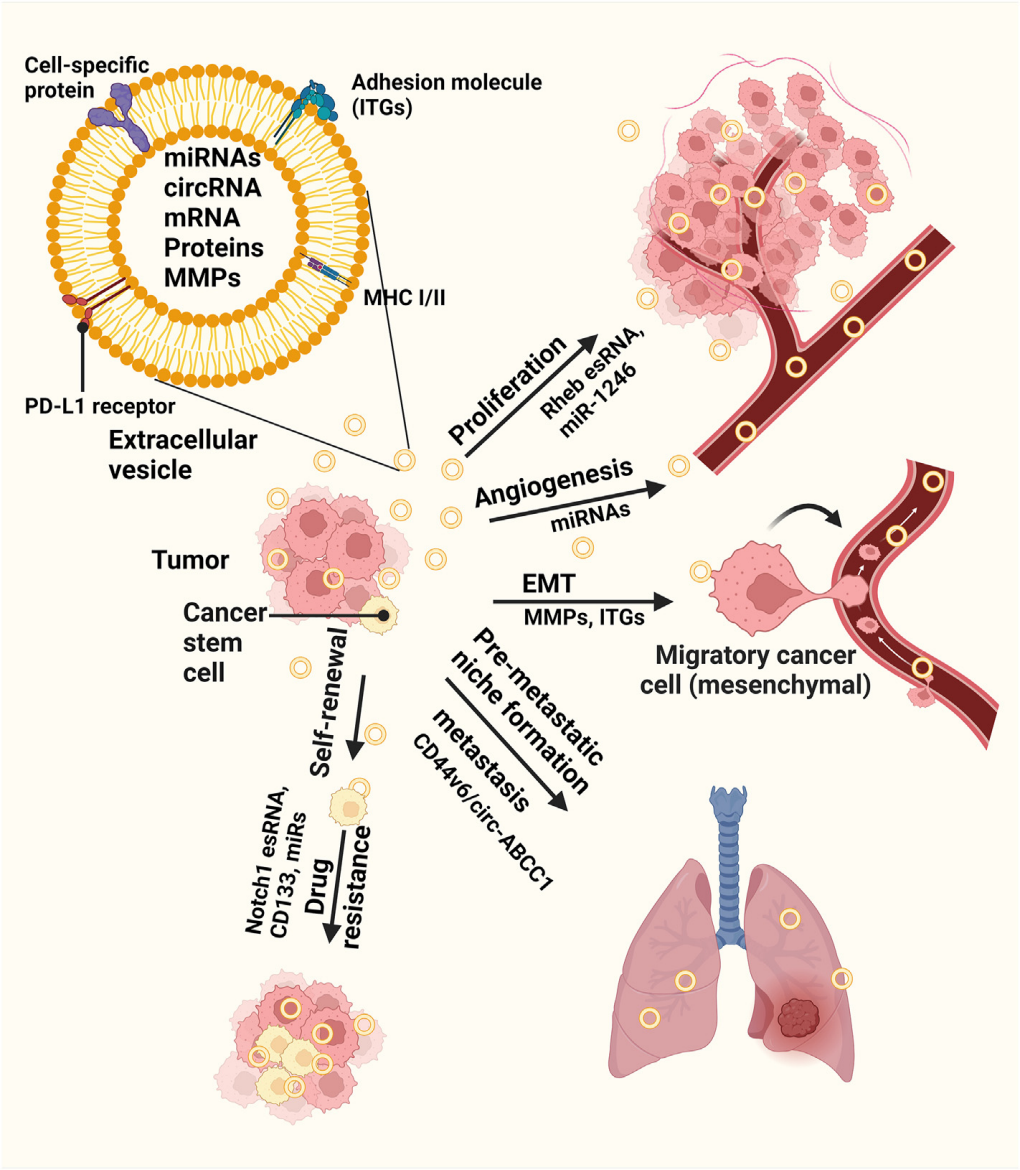
Tumor EV cargo molecules attenuate tumor remodeling. EVs carry proteins, mRNAs, miRNAs, and MMPs that modulate tumors in multiple ways, e.g., by affecting tumor cell proliferation, angiogenesis, EMT, metastasis, and drug resistance. CD: Cluster of differentiation; circ-ABCC1: Circular adenosine triphosphate binding cassette subfamily C member 1; circRNA: Circular RNA; EMT: Epithelial–mesenchymal transition; esRNA: Exosomal shuttle RNA; ITG: Integrin; MHC: Major histocompatibility complex; miR: MicroRNA; miRNA: MicroRNA; MMP: Matrix metalloprotein; mRNA: Messenger RNA; Notch1: Neurogenic locus notch homolog protein 1; PD-L1: Programmed cell death ligand-1; Rheb: A Ras homolog enriched in the brain.

Uncontrolled tumor cell proliferation is associated with abnormal cell-cycle progression. Rheb plays a central role in the mechanistic target of rapamycin (mTOR)-mediated cell proliferation. Rheb, a GTPase, directly interacts with FK506-binding protein 38 (FKBP38), an endogenous inhibitor of mTOR.^4^ Previous data suggested that EVs secreted from Rheb-overexpressing cancer cells contained Rheb esRNAs that could be transferred to neighboring cells and activate the mTOR pathway in recipient cells, based on phosphorylation of the downstream target S6 protein.^2^ These findings suggest that exosomal materials (cargo molecules) can affect the proliferation of surrounding cells [Figure 2].

Li et al.^5^ provided additional evidence that EVs contain miRNAs, specifically microRNA (miR)-1246, which was expressed at higher levels in metastatic breast cancer cells (MDA-MB-231) than in non-metastatic breast cancer or non-malignant breast cells. These EVs transfer miR-1246 to recipient cells and thereby suppress Cyclin*-*G2 *(CCNG2)*, a tumor-suppressor gene that regulates cell proliferation. Decreased *CCNG2* expression serves as a marker of poor prognosis for several types of cancer.^5^ Non-small cell lung cancer (NSCLC) cell-derived EVs promoted cell proliferation and inhibited apoptosis in both normal lung fibroblasts and NSCLC cells by delivering alpha-smooth muscle actin (αSMA).^6^ Moreover, Wang et al.^7^ found that Lovo colorectal cancer (CRC) cells took up Lovo-derived EVs more easily than normal colon epithelial cell (NCM460)-derived EVs, most likely because of the paired cell tropism. Lovo-derived EVs triggered a robust increase in the phosphorylation levels of mitogen-activated protein kinase/extracellular signal-regulated kinase (ERK) kinase (MEK) (p-MEK) and ERK (p-ERK) in a dose-dependent manner, leading to a significant increase in tumor sizes in a xenograft tumor model.^7^

### Modulation of the invasive, migratory, and epithelial–mesenchymal transition properties of cancer cells by extracellular vesicles

The EMT is characterized by the progressive loss of epithelial cells and acquisition of a mesenchymal phenotype, which leads to enhanced migration, invasion, resistance to apoptosis, and extracellular matrix (ECM)-component production. The EMT is not often regulated due to the abnormal activation of transcription factors, expression of specific cell-surface proteins, reorganization, and expression of cytoskeletal proteins, production of ECM-degrading enzymes, and expression of specific miRNAs. EVs released from CSCs carry transcription factors, miRNAs, and cell-adhesion proteins.

Sun et al.^8^ demonstrated that miR-335-5p was more highly expressed in EVs from metastatic SW620 cells than in EVs derived from primary SW480 cells. miR-335-5p, which was transmitted from metastatic SW620 cells to CRC cells via EVs, promoted the migration, invasion, and EMT of CRC cells. Moreover, those EVs expressed vimentin and E-cadherin, which are associated with increased tumor cell invasion and migration. The transmitted miRNA-335-5p decreased RAS p21 protein activator 1 (RASA1) expression and increased Ras protein expression, leading to CRC cell invasion and metastasis by facilitating the EMT.^8^

Gaballa et al.^9^ showed that castration-resistant prostate cancer (CRPC) cell EVs were enriched for integrin A2 (ITGA2), a well-known cell-adhesion molecule. The exosomal transfer of ITGA2 increased Vimentin and focal adhesion kinase (FAK) (EMT markers) expression in recipient cells, resulting in increased CRPC cell migration, as determined in wound-healing and cell-adhesion assays. This was further confirmed by studying ITGA2 expression in plasma samples from patients with prostate cancer (PCa).^9^ Hoshino et al.^10^ analyzed the biodistributions of tumor-secreted EVs and found that EV-ITGs direct organ-specific colonization by fusing with target cells in a tissue-specific manner. Their data revealed that EV-ITGα6, EV-ITGβ4, and EV-ITGβ1 were associated with lung tropism whereas EV-ITGαv and EV-ITGβ5 were showed liver tropism [Figure 2]. In addition, EV-ITGβ3 was produced by brain-tropic cells, indicating that ITGs are selectively packaged into EVs and tend to underlie organotropism in the lungs, liver, and brain. These findings have strong clinical significance because they can predict organ-specific metastasis.^10^

Matrix metalloproteases (MMPs) play vital roles in ECM remodeling, tumor growth, migration, invasion, angiogenesis, and immune suppression. Intriguingly, MMPs have been identified as EV cargo proteins. These EV-associated MMPs control ECM remodeling and receptor shedding from EV membranes or the surfaces of target cells.^11^ EV-associated MMPs participate in stromal cell activation, angiogenesis, and premetastatic niche formation.^11^ Table 1 summarizes the roles of EV-associated MMPs and other cargos associated with EMT. These observations indicate that MMPs associated with EVs can be used as biomarkers of disease progression and responsiveness to anticancer treatments.

**Table 1:** Roles of EV-associated cargo molecules in different cancer events.

Function	Cargo molecule	EV source	Mechanism	Importance	Reference
Cell growth	MMP3	LuM1 tumor cells	Increased tumoroid formation	Cell proliferation in tumoroids and delayed necrosis	^12^
EMT	MMP1	Highly metastatic pulmonary variant of parental MDA-MB-231 cells (MDA-MB-231-HM)	MMP-1 binding to PAR1 promotes triple-negative breast cancer metastasis, possibly via the EMT	Promotes the EMT	^13^
	miR-19-3p	CCRCCs	Decreased PTEN expression	Promotes the EMT and lung metastasis	^14^
	miR-155	Breast CSCs	TGF-β, C/EBP-β, and FOXO3a repression	Enhances chemoresistance, promotion of the EMT	^15^
	miR-375-3p	Colon cancer CSCs	Reduced expression of β-catenin, vimentin, and SNAIL	EMT suppression, a potent therapeutic agent for the treatment	^16^
	ALDOA/ALDH3A1	Lung cancer	Accelerated glycolysis	Enhances migration and invasion	^17^
Angiogenesis	MMP14	Corneal fibroblasts	MMP2 recruitment	Corneal angiogenesis	^18^
	miR-21	Glioma stem cells (GSCs)	VEGF-VEGFR2 signaling pathway	Promotion of angiogenesis	^19^
	miR-26a	GSCs	PTEN-PI3K-Akt pathway	Promotion of angiogenesis	^20^
	EPHB2	HCC cells	Induced ephrin-B reverse signaling through STAT3	Induction of tumor angiogenesis	^21^
Metastasis	MMP9	High-grade serous ovarian cancer (HGSOC)	Distinct presence of annexin V-binding EVs	Increases malignancy, can potentially serve as an HGSOC biomarker	^22^
	MMP13	Nasopharyngeal cancer (NPC)	Increased EMT in NPC cells	Increases tumor cell metastatic properties	^23^
	CEMIP	Head and neck squamous cell carcinoma	Upregulation of pro-inflammatory cytokines encoded by Ptgs2, Tnf, and Ccl/Cxcl	Cancer cell colonization in brain metastasis	^24^
	Annexin II	Breast cancer	Activation of the P38mapk, NF-κB, and STAT3 pathways	Promotes breast cancer metastasis to lungs	^25^
Cancer stemness	miR-146a-5p	Colorectal cancer stem cells (CRCSCs)	Decreased numb expression	Promotes CSC stemness and a tumor-immunosuppressive microenvironment	^26^
	miR-454	Breast CSCs	Activation of the PRRT2-Wnt pathway	Promotes CSC stemness	^27^
Drug resistance	miR-21-5p	OSCC stem cells	Activation of the STAT3-mTOR-β-catenin pathway	Increases tumorigenicity and chemo-resistance	^28^
					^26^
	Annexin A6	Gastric cancer	Activation of β1 integrin-FAK-YAP pathway	Attenuates gastric cancer drug resistance	^29^
	miR-210	Human pancreatic cancer stem cells	mTOR activation	Gemcitabine-based chemotherapy resistance	^30^

Akt: Protein kinase B; ALDH3A1: Aldehyde dehydrogenase 3 family member A1; ALDOA: Aldolase, fructose-bisphosphate A; Ccl: C–C motif chemokine ligand; CCRCC: Clear cell renal cell carcinoma stem cell; C/EBP-β: CCAAT enhancer binding protein beta; CEMIP: Cell migration-inducing hyaluronan-binding protein; CRCSC: Colorectal cancer stem cell; CSC: Cancer stem cell; Cxcl: C–X–C motif chemokine ligand; EMT: Epithelial–mesenchymal transition; EPHB2: EPH receptor B2; EV: Extracellular vesicle; FAK: Focal adhesion kinase; FOXO3a: Forkhead box O3; GSC: Glioma stem cell; HCC: Hepatocellular carcinoma; HGSOC: High-grade serous ovarian cancer; LuM1: lung-metastatic1 cells; MDA-MB-231: Triple-negative Breast cancer cells; MDA-MB-231-HM: Highly metastatic triple negative breast cancer cells; miR: MicroRNA; MMP: Matrix metalloprotein; mTOR: Mechanistic target of rapamycin; NF-κB: Nuclear factor-kappa B; NPC: Nasopharyngeal cancer; OSCC: Oral squamous cell carcinoma; P38mapk: P38 mitogen-activated protein kinase; PAR1: Protease-activated receptor-1; PI3K: Phosphoinositide 3-kinase; PRRT2: Proline-rich transmembrane protein 2; PTEN: Phosphatase and tensin homolog; Ptgs2: Prostaglandin-endoperoxide synthase; SNAIL: Snail family transcriptional repressor 1; STAT3: Signal transducer and activator of transcription 3; TGF-β: Transforming growth factor-beta; Tnf: Tumor necrosis factor; VEGF: Vascular endothelial growth factor; VEGFR2: Vascular endothelial growth factor receptor 2; Wnt: Wingless-related integration site; YAP: Yes1 associated transcriptional regulator.

### Extracellular vesicle cargo molecules and cancer-stemness properties during tumor growth and metastasis

CSCs are considered responsible for tumor progression, drug resistance, and disease relapse. Furthermore, CSC-derived EVs carry multiple bioactive molecules that promote tumor cell EMT, tumor angiogenesis, vascular permeability, drug resistance, and immune suppression, which significantly contribute to cancer metastasis. CSC-derived EV cargo molecules activate multiple cell-fate pathways such as Notch1, Wingless-related integration site (Wnt)-β-catenin, and receptor tyrosine kinase (RTK)-G protein-coupled receptor (GPCR)-ITG pathways. For example, the Notch1-signaling pathway is a well-conserved intercellular communication network that regulates cell differentiation and cell fates. Notch1 also plays a critical role in maintaining cancer cell stemness. Upon activation, Notch1 is cleaved and the intracellular domain of Notch (ICN) is released, after which it enters the nucleus and recruits transcriptional activators to the CBF1, Suppressor of Hairless, or Lag-1 (CSL) complex. This complex converts a transcriptional repressor into an activator that activates Notch target genes such as *Hes-1*, Nuclear factor-kappa B (*NF-κB*), *Cyclin D1*, and *c-Myc*. Recent findings showed that angiomyolipoma (AML) tumor cell-derived EVs contain NOTCH1 esRNAs, leading to constitutive activation of the Notch1 pathway. Constant Notch1 activation caused the cell-differentiation profiles to change toward multilineage progenitors, suggesting a role for exosomal *esNotch1* in maintaining cancer cell fate and differentiation.^31^

Expression of the well-established CSC marker, cluster of differentiation (CD)133, is modulated by pancreatic and colorectal CSCs. Recently, Zhao et al.^32^ identified and amplified the linear isoform of circular adenosine triphosphate binding cassette subfamily C member 1 (circ-ABCC1) from both complementary DNA (cDNA) and genomic DNA (gDNA) in EVs from CD133^+^ Caco2 cells and HCT15 cancer cells. The uptake of circ-ABCC1 containing EVs mediated β-catenin entry into the nucleus of CD133^+^ cells, resulting in Wnt-pathway activation and increased cancer stemness and metastasis in CRC,^32^ as represented in Figure 2. Another important factor associated with cancer stemness and cancer development/metastasis is CD44, including both its standard (CD44s) and variant (CD44v) forms. CD44 associated with tumor-derived EVs has been linked to cancer progression, particularly premetastatic niche formation and metastasis. CD44 and CD44 variants have been observed on tumor EVs derived from gastric,^33^ ovarian,^34^ and pancreatic^35^^,^^36^ cancer cell lines and to cause tumorigenesis and metastasis.

The versatility of EV cargo molecules in different CSCs is an important factor in tumor malignancy and disease spread to distant organs. EV cargo biomolecules contain both proteins and different miRNAs that help recipient cells activate transcriptional pathways. For example, in a study of clear cell renal cell carcinoma stem cells (CCRCCs), *Wang* et al.^14^ reported that miR-19b-3p was transmitted to cancer cells via CSC-EV-induced EMT, leading to repressed phosphatase and tensin homolog (PTEN) expression. In addition, oral squamous cell carcinoma (OSCC) stem cell-derived EVs contain miR-21-5p, which activates the phosphoinositide 3-kinase (PI3K)-mTOR-signal transducer and activator of transcription 3 (STAT3) signaling pathway in OSCC cells, leading to cisplatin resistance in non-OSCC stem cells. CSC-EVs confer resistance to gemcitabine-sensitive pancreatic cancer cells by delivering miR-210^30^ [Figure 2]. Interestingly, breast cancer cell (MDA-MB-231)-derived EVs carry miR-454 and disrupt the Wnt pathway by targeting proline-rich transmembrane protein 2 (PRRT2), thereby promoting CSC stemness and ovarian cancer cell growth *in vivo*.^27^ The details of the presence and activity of EV cargo molecules are summarized in Table 1.

### Systemic circulation of tumor extracellular vesicles involved in pre-metastatic niche formation

The pre-metastatic niche establishes a favorable microenvironment in secondary organs for subsequent tumor metastasis in the presence of a primary tumor. Pre-metastatic niche formation is influenced by several bodily processes, such as immune system suppression, increased levels of cytokines and other growth factors, ECM deposition and remodeling, hypoxia in the primary tumor, and movement of EVs from a primary tumor to secondary organs.

Fang et al.^37^ reported that highly metastatic hepatocellular carcinoma (HCC) cells exhibited a greater capacity to convert normal fibroblasts to cancer-associated fibroblasts (CAFs) in the lungs, creating a pre-metastatic niche. Mechanistic data revealed that highly metastatic HCC cells secreted exosomal miR-1247-3p, both *in vitro* and in patients, which directly targeted B4GALT3, leading to ITGβ1-NF-κB-signaling activation in fibroblasts. Activated CAFs can promote cancer progression by secreting pro-inflammatory cytokines, including interleukin (IL)-6 and IL-8.^37^ By collecting more evidence on the role of EVs in a premetastatic niche, Xie et al.^38^ provided details regarding pancreatic ductal adenocarcinoma (PDAC)-derived EVs carrying another stem cell marker complex, i.e., the CD44v6-C1QBP complex. Delivery of this complex to the plasma membrane of hepatic satellite cells (HSCs) can lead to the phosphorylation of insulin-like growth factor 1 signaling molecules, which results in HSC activation and liver fibrosis. A clinical study has shown that EV CD44v6 and C1QBP expression was higher in patients with PDAC and liver metastasis than in those without liver metastasis. In addition, simultaneous high expression of exosomal CD44v6 and C1QBP correlated with a worse prognosis and a higher risk for postoperative PDAC liver metastasis.^38^

## Effect of extracellular vesicles in immune homeostasis and tumor-immune microenvironment

### Tumor extracellular vesicles regulate the immune response

**Figure 3:**
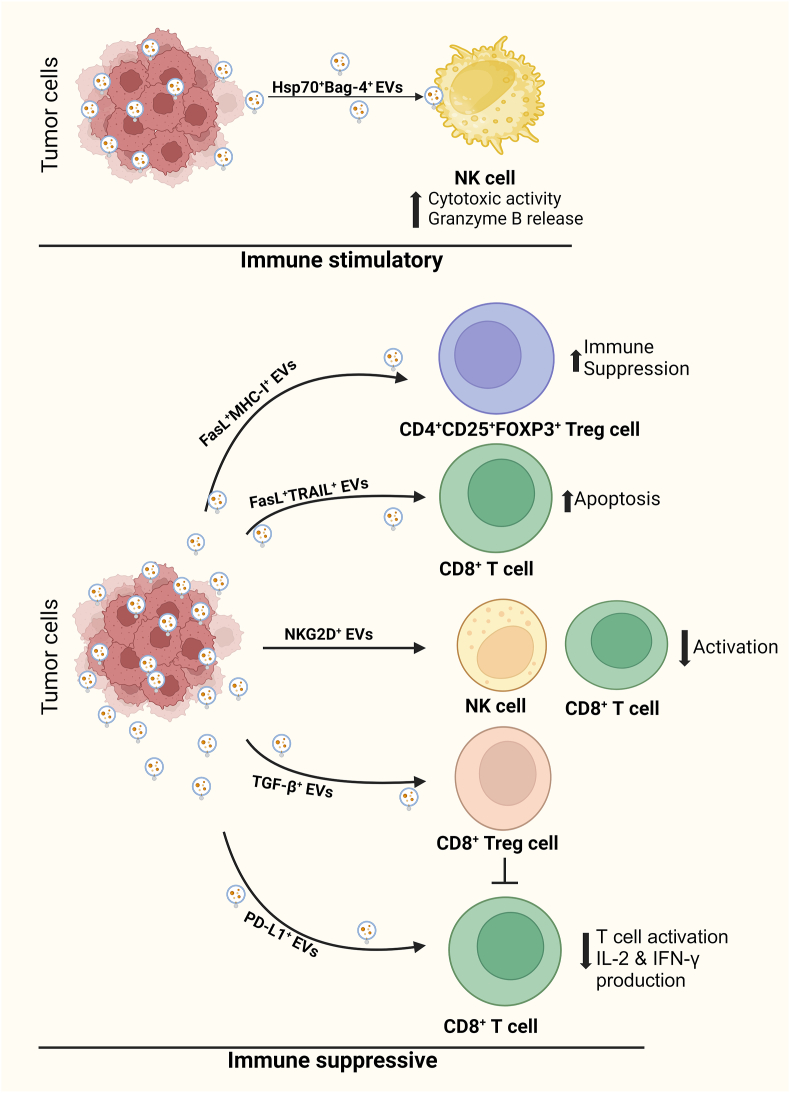
Role of EVs in the immune TME. EV cargo molecules can change the TME in a stimulatory or suppressive manner, although they function as immune suppressors. EVs secreted from tumor cells carry PD-L1, TGF-β, FasL, TRAIL, or MHC I/II, which suppress the functions of CD8^+^ T cells and NK cells and help establish immune homeostasis. Bag-4: Bcl-2-associated athanogene-4; CD: Cluster of differentiation; EV: Extracellular vesicle; FasL: Fas ligand; FOXP3: Forkhead box P3; Hsp70: Heat shock protein 70; IL-2: Interleukin 2; INF-γ: Interferon-gamma; MHC: Major histocompatibility complex; NK: Natural killer; NKG2D: Natural killer group 2, member D; PD-L1: Programmed cell death ligand-1; TGF-β: Transforming growth factor-beta; TME: Tumor microenvironment; TRAIL: Tumor necrosis factor-related apoptosis-inducing ligand; Treg: T regulatory.

Tumor EVs play essential roles in remodeling the tumor immune microenvironment even before the occurrence and metastasis of cancer. Tumor-secreted EVs modulate immune responses through both immunostimulatory and immunosuppressive pathways. Tumor-derived EVs bearing heat shock protein 70 (HSP70) promote migratory and cytolytic activities of natural killer (NK) cells and increase tumor necrosis factor-alpha (TNF-α) production in macrophages, thus creating an immune-stimulatory environment and providing anti-tumor immunity.^39^ However, tumor-associated EVs can promote immunosuppressive and pro-tumorigenic effects. For example, programmed cell death ligand-1 (PD-L1) and PD-L2 (B7DC and CD273) are ligands of programmed cell death protein-1 (PD-1), an immune checkpoint receptor that is expressed on the surface of T cells. Blocking the PD-1-PD-L1 pathway is a promising new form of cancer therapy. The results of several recent studies have identified exosomal PD-L1 in the blood of patients with various cancers, including head and neck cancer, melanoma, and NSCLC. Kim et al.^40^ showed that EVs derived from lung cancer cells expressed PD-L1 and played a role in immune escape by reducing T cell activity and promoting tumor growth. The abundance of PD-L1 in EVs represents the level of PD-L1 expression on the cell surface. After administering PD-L1-containing EVs, decreased IL-2 and IFN-γ production fewer total CD8^+^ T cells have been found [Figure 3]. This PD-L1-mediated immune response promoted tumor growth.^40^ Furthermore, melanoma patients resistant to anti-PD-1 therapy had elevated levels of exosomal PD-L1 in their blood before treatment.

In addition to PD-L1, NSCLC-derived EVs had higher expression of the immune checkpoint proteins Tim-3 and Galectin-9, which correlated positively with several malignant parameters, including larger tumor size, an advanced disease stage, and more distant metastasis.^41^ Another immune checkpoint protein, cytotoxic T-lymphocyte-associated protein 4 (CTLA-4), was identified in HCC-derived EVs. CTLA-4 regulates the PTEN-CD44 signaling pathway to promote the invasion and metastasis of HCC.^42^

EVs derived from prostate and ovarian cancer express Fas ligand (FasL, also known as CD95L) and tumor necrosis factor-related apoptosis-inducing ligand (TRAIL) (which induces T cell apoptosis), respectively; thus, they act as antigen-presenting death signals for CD8^+^ T cells.^43^ Transforming growth factor-beta (TGF-β) was transmitted via breast cancer EVs under hypoxic conditions which suppressed T cell proliferation.^44^ EVs produced by various cancer cell lines *in vitro* or isolated from pleural effusions from patients with mesothelioma carry natural killer group 2 (NKG2D) ligands that downregulate the surface expression of NKG2D in NK and CD8^+^ T cells, which prevent NK and CD8^+^ T cell activation.^45^ Additionally, Whiteside et al.^46^ demonstrated the effect of tumor-derived EVs on CD4^+^ T regulatory (Treg) cells. CD4^+^ T cells pre-activated with an anti-CD3 antibody and cultured with tumor cell-derived EVs induced a substantial expansion of CD4^+^CD25^+^ forkhead box P3 (FOXP3^+^) Tregs.^46^ Tregs comprise a subset of potent immunosuppressive cells that play vital roles in maintaining immune homeostasis. Thus, tumor EVs can cause immune suppression by delivering immune checkpoint inhibitors, inducing T cell apoptosis, and promoting Treg differentiation.

### Modulating of tumor cell-stromal cell crosstalk in the tumor microenvironment by extracellular vesicles

In the TME, communication between stromal and tumor cells plays an important role in the occurrence and development of tumors. Tumor stromal cells include multiple forms of tumor-infiltrating immune cells (such as myeloid-derived suppressor cells [MDSCs], B cells, T cells, and tumor-associated macrophages [TAMs]), tumor-associated endothelial cells, CAFs, and tumor-associated adipocytes. Stefanius et al.^47^ provided evidence that incubating pancreatic tumor cell EVs with NIH/3T3 fibroblasts initiated the formation of foci. Their *in vivo* data confirmed that the EV-exposed fibroblasts grew into tumors. These results demonstrate that cancer EVs transform normal fibroblasts into CAFs.^47^ Furthermore, EV-mediated delivery of TGF-β led to the suppressor of mothers against decapentaplegic (SMAD)-dependent fibroblast activation, including αSMA and fibroblast growth factor 2 (FGF2) expression in fibroblasts and triggered fibroblasts to undergo myofibroblast differentiation.^48^ In addition to CAFs in the TME, EVs modulate tumor-infiltrating immune cells, mainly TAMs and MDSCs. TAMs regulate multiple fundamental processes during neoplastic pathogenesis and traditionally act as antitumor-polarized M1 macrophages. The results of numerous studies have indicated that TAMs undergo a polarization shift from an antitumor M1 phenotype to a pro-tumor M2 phenotype in response to cancer EVs,^49^ although the underlying mechanism remains unclear. In addition to TAMs, tumor EVs enhance the immunosuppressive functions of MDSCs. Intriguingly, the exosomal transfer of miR-9 and miR181a activates SOCS3, leading to activation of the Janus kinase (JAK)-STAT pathway in MDSCs and enhanced tumor-suppressive function in breast cancer.^50^ Gao et al.^51^ also reported that renal cancer-derived EVs carried HSP70, which can induce tumor immune tolerance via MDSC-mediated antigen-specific immunosuppression. Compiling current data provides sufficient proof that (1) interactions between tumor-derived EVs and stromal cells create an immunosuppressive microenvironment that facilitates tumor development and resistance to tumor therapy and that (2) inhibiting tumor-derived EV production or injecting bioengineered EVs to target the TME can improve anti-cancer drug responses.

## Extracellular vesicles as biomarkers for cancer diagnosis

Noninvasive biopsies (blood or body-fluid biopsies) have attracted substantial interest, mainly in the context of diagnosing tumors or metastasis and assessing treatment responses. Tumor-derived EVs carry distinct cancer proteins that serve as biomarkers for tumor progression, metastasis, and immune evasion. EVs have been investigated for almost all types of cancer. EVs derived from the serum of patients with pancreatic cancer showed high glypican-1 (GPC1^+^) levels, which correlated with the tumor burden and survival of patients before and after surgical tumor resection.^52^ Interestingly, cells harboring gDNA mutations released EVs containing DNA bearing the same mutations. This phenomenon has been observed in various settings, such as in cell culture supernatants, plasma from tumor-bearing mice, and blood (serum and plasma) from individuals with cancer.^53^ Kahlert et al.^54^ demonstrated that EVs from human serum samples contained gDNA spanning all chromosomes and contained gDNA with *KRAS* and *p53* mutations. These results indicate that serum-derived EVs can be used to detect crucial gDNA mutations relevant to cancer prediction, treatment, and therapy resistance.^54^ In addition, Allenson et al.^55^ found a high prevalence of mutant KRAS DNA in circulating EVs from the plasma of patients with early-stage pancreatic cancer. Thakur et al.^56^ demonstrated the presence of double-stranded gDNA in exosomes, representing the entire genome. Next-generation sequencing of plasma EV-derived nucleic acids from 41 patients showed a high detection rate (∼95%) for common BRAF, KRAS, and epidermal growth factor receptor (*EGFR*) mutations, surpassing the sensitivity of clinical testing with archival tumors or cell-free plasma gDNA. Together with these findings, patient survival data indicated that detecting mutations in EV-derived nucleic acid with a low mutation allelic frequency (MAF) is an important prognostic factor for longer survival. EVs isolated from patient plasma samples carried the *EGFR* T790M mutation associated with NSCLC. Detecting the T790M mutation in EV nucleic acids enabled 92% sensitivity and 89% specificity with tumor biopsies.^57^ Thus, performing a detailed analysis of proteins, DNA, and RNA extracted from EVs shed by tumors in patient biofluids holds promise as a clinical method for diagnosing cancer, categorizing patients for tailored therapies, and overseeing treatment progress. This approach eliminates the need for obtaining tumor samples directly.

## Extracellular vesicles, A therapeutic tool for cancer treatment

EVs enable intricate and sophisticated cellular communication. Through EVs, cells share information in the form of lipids, proteins, or nucleic acids. Their ability to transfer biomolecules makes EV an excellent choice for use in therapy. When developing EV-based therapies, the primary aspect to consider is the cellular origin of EV. Consequently, EVs derived from inflammatory cells inherently serve distinctly different biological roles than those originating from mesenchymal stromal cells (MSCs). To date, multiple efforts have been made to develop MSC-EVs as therapeutic tools and several experiments have shown that EVs originating from MSCs replicate the immunoregulatory capabilities and regenerative potential of MSCs.

Currently, anti-cancer therapeutics involve a much lower standard of care (*vs.* therapeutics for other disease types) due to urgent and unmet medical needs, suggesting that room exists for an effective EV-based therapeutic that could substantially improve patient care. The first clinical trial involving EV administration to patients was performed in 2005 for melanoma. In that trial,^58^ EVs from dendritic cells (DCs) containing antigen-presenting molecules (major histocompatibility complex [MHC] class I, class II, CD1, and hsp70–90), tetraspanin molecules (CD9, CD63, and CD81), adhesion molecules (CD11b and CD54), and CD86 co-stimulatory molecules were administered to patients with melanoma. Although one of 15 patients exhibited a melanoma antigen recognized by T cells 1 (MART1)-specific, human leukocyte antigen (HLA)-A2 restricted T cell response in the tumor bed associated with the progressive loss of HLA-A2 and HLA-BC on tumor cells during therapy, the results demonstrated the feasibility of large-scale EV production and the safety of EV administration. *In vivo* data showed that introducing DC-derived EVs eradicated the growth of established murine tumors in a T cell-dependent manner.^59^ Morse et al.^60^ tested the safety, feasibility, and efficacy of autologous DC-derived exosome (DEX) loaded with melanoma antigen gene (*MAGE*) tumor antigens in patients with NSCLC. The patients received four doses of DEX at weekly intervals. All doses were well tolerated with minor events; thus, the dosing was feasible. Some patients experienced long-term disease stability and activation of immune effectors.^60^ Furthermore, administering ascites-derived exosome (Aex) in combination with granulocyte-macrophage colony-stimulating factor (GM-CSF) was used for CRC therapy. The result showed that Aex plus GM-CSF, but not Aex alone, induced a beneficial tumor-specific antitumor cytotoxic T lymphocyte (CTL) response.^61^ Additional clinical trials for cancer therapy involving conducted EVs are presented in Table 2. Moreover, clinical trials are currently underway to explore the potential of plant EVs (PEVs) to deliver drugs more effectively to tumors.

**Table 2:** Clinical trials of exosome-based therapy for cancer.

Clinical trial ID	EV source	Cancer type	Status	Clinical objective
NCT03608631	MSC-derived exosomes loaded with KRASG12D siRNA	Metastatic pancreatic cancer	Recruiting	To identify the maximum tolerated dose of MSC-derived exosomes loaded with siRNA against *Kras*G12D (iExosomes) in patients with metastatic PDAC and the *Kras*G12D mutation
NCT02393703	Blood and tissue	Pancreatic cancer	Recruiting	To interrogate exosome-mediated intercellular signaling in patients with pancreatic cancer
NCT02147418	Human papillomavirus-positive OSCC	Oropharyngeal squamous cell carcinoma	Ongoing	To study exosome testing as a screening modality for human papillomavirus-positive oropharyngeal squamous cell carcinoma
NCT04529915	Plasma	Lung cancer	Active, not recruiting	To diagnose lung cancer at an early-stage using blood plasma-derived exosomes
NCT02869685	Plasma	Advanced NSCLC	Completed	To analyze the consistency of PD-L1 expression in advanced NSCLC tissues and in plasma exosomes before and after radiotherapy
NCT05286684	Cerebrospinal fluid	Metastatic meningitis from breast cancer	Ongoing	To assess the feasibility of exosome analysis for cerebrospinal fluid during the diagnostic workup of metastatic meningitis from patients with breast cancer
NCT04939324	Patients with early-stage lung cancer	Early-stage lung cancer	Ongoing	To molecularly profile exosomes in tumor-draining veins of patients with early-stage lung cancer
NCT03738319	Epithelial ovarian cancer	HGSOC	Completed	To analyze miRNA and lncRNA expression in patients with HGSOC and benign gynecologic diseases via next-generation sequencing
NCT02702856	Urine	Prostate cancer	Completed	To clinically validate a urinary exosome gene signature in men with suspected prostate cancer
NCT03032913	GPC1^+^ exosomes from plasma samples	Pancreatic cancer	Completed	To determine whether liquid-biopsy approaches are valid for diagnosing pancreatic cancer

EV: Extracellular vesicle; GPC: Glypican 1; HGSOC: High-grade serous ovarian cancer; ID: Inhibitors of DNA binding and cell differentiation; KRAS: Kirsten rat sarcoma virus; KRASG12D: Kirsten rat sarcoma virus G12D form; lncRNA: Long noncoding RNA; miRNA: MicroRNA; MSC: Mesenchymal stem cell; NSCLC: Non-small lung cell carcinoma; OSCC: Oral squamous cell carcinoma; PDAC: Pancreatic ductal adenocarcinoma; PD-L1: Programmed cell death ligand-1; siRNA: Small-interfering RNA.

### Plant extracellular vesicles for cancer therapy

Understanding the biological characteristics and functions of PEVs has provided new drug-delivery platforms with clinical applications. Similar to mammalian EVs, PEVs contain numerous proteins, lipids, and miRNAs that act as cellular messengers that transfer these biologically active substances. The abundance and relatively easy isolation of PEVs have also been considered encouraging for cancer treatment. Stanly et al.^62^ demonstrated that PEVs from lemons, grapefruits, and oranges inhibited the proliferation of lung, skin, and breast cancer cells but not normal cells. In particular, grapefruit PEVs arrested tumor cells at the G2/M phase of the cell cycle and reduced Cyclin B1/B2 expression.^62^ As another example, garlic PEVs induced cell cycle arrest in the S phase and activated caspase-mediated apoptosis in renal and lung cancer cells.^63^ Ginseng EVs promoted the transformation of TAMs from the M2 phenotype to the M1 phenotype and inhibited melanoma growth in a manner that depended on Toll-like receptor 4 (TLR4) and myeloid differentiation primary response protein 88 (MyD88) signaling.^64^ Asparagus PEVs inhibited the proliferation of liver cancer cells by reducing antigen Kiel 67 (Ki67) and proliferating cell nuclear antigen (PCNA) expression and increasing the expression of apoptosis-inducing factor (AIF), Bax, and Bak (which trigger caspase-9 and poly(adenosine diphosphate-ribose) polymerase [PARP] cleavage in liver cancer cells *in vitro* and *in vivo*).^65^ PEV-based cancer therapy is a promising avenue for cancer treatment and two early-phase clinical trials are currently ongoing. In clinical trial NCT01668849, grape PEVs were used to prevent oral mucositis, a common side effect associated with chemotherapy for head and neck cancer. That trial was designed to include the influence of grape PEVs on cytokine production, immune responses to tumor exosomal antigens, and metabolic and molecular markers in patients. In clinical trial NCT01294072, the ability of PEVs to deliver curcumin to normal and colon cancer tissues was studied. The results of both trials indicated that PEVs were safe as therapeutic tools and that further exploration in this area is required.

### Bio-engineering extracellular vesicles

EVs are relatively non-immunogenic, biodegradable, and biocompatible and can cross the blood–brain barrier. Moreover, EVs can be bioengineered to carry therapeutic molecules such as proteins, RNAs, or DNAs. Furthermore, many studies are ongoing to optimize their therapeutic potential by increasing EV production and release from producer cells, improving the cargo-loading efficiency, increasing the tissue tropism, and boosting cargo release into target cells.^66^

**Figure 4:**
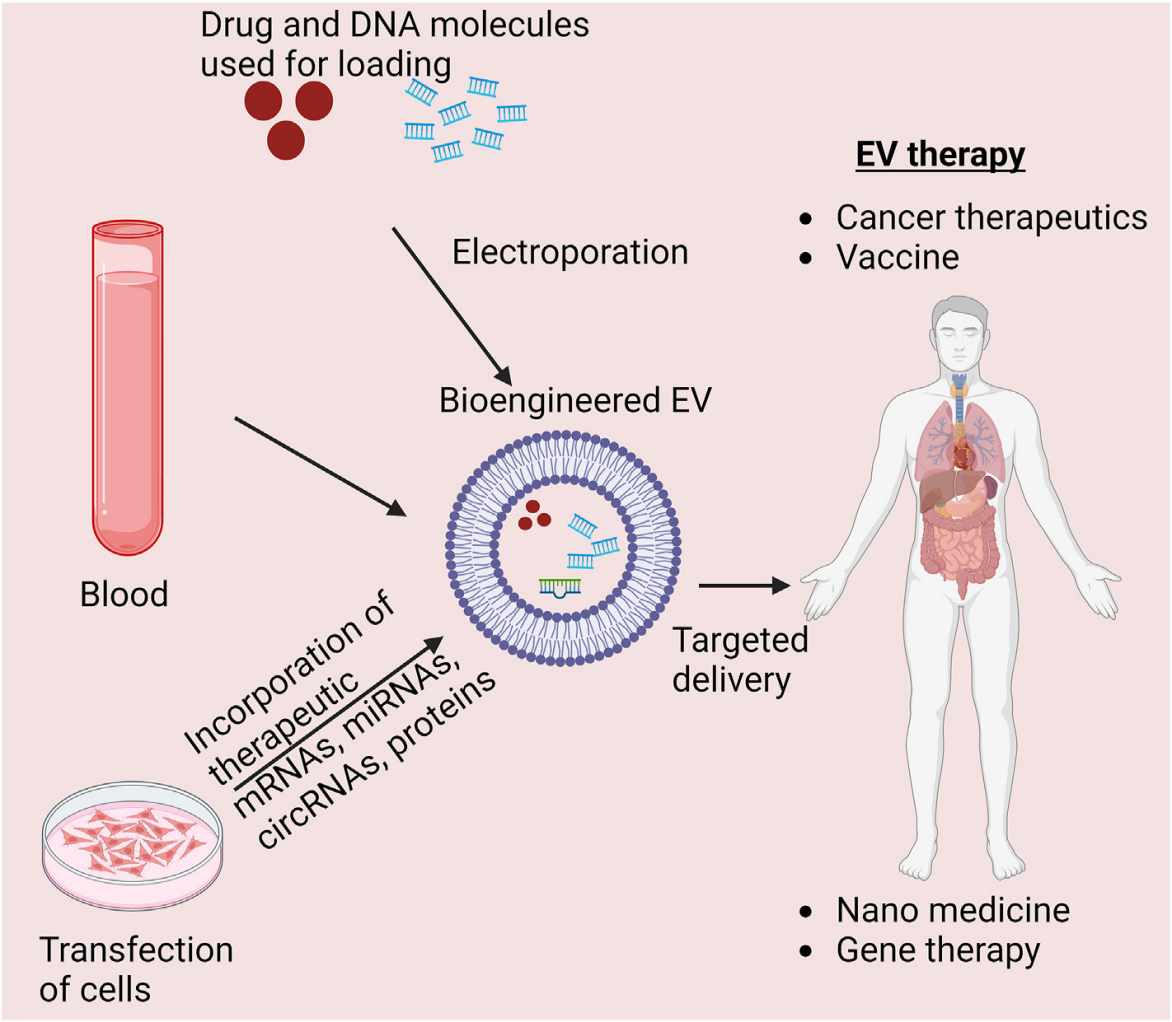
Bioengineering EVs for therapeutic applications. circRNA: Circular RNA; EV: Extracellular vesicle; mRNA: Messenger RNA; miR: MicroRNA.

Although EVs show great potential as promising delivery platforms, the effective incorporation of therapeutic cargo molecules into EVs poses significant challenges. The loading of therapeutic materials into EVs can occur either naturally during their formation or after the EVs have been isolated using various physical or chemical techniques [Figure 4]. Electroporation is commonly used to introduce nucleic acids into EVs, although it can adversely affect the inherent properties of the EV membrane, resulting in substantial EV loss. Therefore, the prevailing method for loading mRNA into EVs involves transfecting EV-producing cells with plasmids encoding therapeutic mRNA. Recently, Villamizar et al.^67^ transfected MSCs with a plasmid encoding a zinc finger transcription factor specifically designed to target the cystic fibrosis transmembrane conductance regulator (*CFTR*) gene promoter. EVs purified from transfected MSCs have sufficient amounts of CFTR mRNA and protein to treat cystic fibrosis.^67^ Greco et al.^68^ utilized EVs as delivery vectors to transport polo-like kinase 1 (PLK-1) siRNA to bladder cancer cells *in vitro*, resulting in selective silencing of PLK-1 and significantly reduced cell proliferation. These findings demonstrate that EVs are potentially useful for intravesical therapy.^68^

miRNAs are widely present within EVs, where they play functional roles in regulating gene expression in various types of recipient cells. miRNAs appear to be preferentially loading into EVs over other types of RNA, indicating the presence of an intrinsic cellular-loading mechanism. O'Brien et al.^69^ successfully engineered adult MSCs to secrete EVs enriched with miR-379 for *in vivo* breast cancer therapy. Following the uptake of miR-379-enriched EVs, miR-379 reduced the expression of cyclooxygenase-2 (COX-2), which is well associated with breast cancer progression and a poor patient prognosis, leading to a decreased tumor burden.^69^ Electroporation is another method used to load miRNAs into EVs. Brossa et al.^70^ demonstrated miR-145 loading into EVs. miR-145^+^ human liver stem cell (HLSC)-derived EVs more effectively inhibited CSC invasion than naïve vesicles.^70^ Due to their profound regulatory potential and natural occurrence in EVs, natural or engineered miRNA incorporation appears to be a promising approach for EV therapeutics.

Circular RNAs (circRNAs) constitute a class of single-stranded, circular, non-coding RNA molecules formed through the back-splicing of mRNA exons. In some instances, circRNAs are produced at levels several times higher than their corresponding protein-coding mRNAs, implying their significant functional roles.^71^ Notably, the circular structure of circRNAs, lacking traditional 5′ and 3′ ends, affords them protection from degradation by exonucleases. Recent data showed that functional circRNAs can be loaded into EVs and subsequently transferred to recipient cells. Interestingly, circRNAs: linear RNA ratio within EVs surpassed that in the producer cells, suggesting the existence of an inherent sorting mechanism responsible for selective circRNAs packaging into EVs. Owing to their increased stability, circRNAs can be packaged into EVs and transferred to target cells, where they may support protein translation for unusually long mRNAs. Qu et al.^72^ designed a circRNA encoding the spike protein of severe acute respiratory syndrome coronavirus 2 (SARS-CoV-2). They subsequently conducted mouse experiments demonstrating the immunization potential of circRNA vaccines encapsulated within lipid nanoparticles (LNPs).^72^ Collectively, these findings imply that circRNAs can generate more protein than linear mRNAs, which could enhance the overall effectiveness of mRNA-based therapies. Consequently, circRNAs may prove valuable for various medical applications, including vaccine development and treating conditions such as cancer, infectious diseases, and genetic disorders. Ideally, therapeutic circRNAs should be engineered to encode specific therapeutic proteins and loaded into EVs. This approach facilitates sustained protein expression within the target cells and offers promise for long-lasting therapeutic benefits.

## Challenges and future prospective

Despite the potential of EV therapeutics, several challenges need to be addressed prior to their clinical implementation.

### Extracellular vesicles heterogeneity

EV heterogeneity poses a significant challenge for EV isolation, characterization, and clinical applications. Mass spectrometry and nanoparticle-tracking analyses suggest that size and compositional heterogeneity are present in EVs derived from various types of cells. EV heterogeneity is not limited to size and composition but also relies on the cellular source, physiological state, and biological environment of cells. Protein and RNA levels and their incorporation into EVs vary stochastically. Stochasticity is primarily responsible for batch-to-batch variations in EVs. However, EVs from different cell types carry unique proteins that are not found in EVs from other cell types.^73^

### Techniques for extracellular vesicle isolation and standardization

Currently, the most popular EV-isolation method is size-based separation. That method has limitations, as it is not completely clear whether EVs affecting certain biogenic pathways are more likely to be in a particular size range. To address this challenge, considerable research effort has been devoted to developing different isolation methods, such as composition- or affinity-based isolation. EV surface proteins such as CD9, CD63, and CD81 have been utilized for composition- or affinity-based separation methods. EV isolation using these methods, which provide a relatively pure population, has yet to be standardized.^74^

### Storage and stability

The International Society for Extracellular Vesicles (ISEV) has shown that storing EVs at −80 °C in phosphate-buffered saline can preserve their size, shape, and cargo molecules.^75^ Several other research groups have reported similar storage conditions.^76^ However, the biological efficacy of other biological fluids has not been thoroughly tested.

### Exosome profiling

Currently, EV profiling is performed using traditional methods such as western blotting or enzyme-linked immunosorbent assay (ELISA), which involve numerous proteins and biomarkers. Moreover, these assays are contaminated because normal cells secrete EVs that interfere with detection. Recently, Ji et al.^77^ developed a microchip platform for single-cell EV profiling and demonstrated the heterogeneity of EVs secreted from single cells. This assay revealed that previously unobserved cellular heterogeneity underlaid EV secretion, which may open new avenues for studying the utility of EV analysis in cancer diagnosis.^77^

### Exosome barcoding

As traditional approaches fail to detect the early stages of cancer, a more specific EV-based screening method (i.e., “exosome barcoding”) should be considered as a solution. Zhang et al.^78^ developed highly sensitive EV barcoding using surface-enhanced Raman spectroscopy nanotags, which showed a greater multiplexing capability and provided an alternative noninvasive liquid-biopsy analysis for cancer diagnosis. Lee et al.^79^ developed a single EV analysis (SEA) technique and identified individual vesicles by staining EVs with fluorophore-labeled streptavidin. Using this technique, they detected EGFRvIII-positive vesicles and isocitrate dehydrogenase 1 (IDH1) R132H^+^ cancer cell-derived EVs. This approach was advantageous because it overcame several challenges by avoiding expensive magnetic-resonance imaging (MRI) and noninvasive cancer liquid biopsies.^79^

## Conclusion

EV vesicles have been extensively studied in the context of cancer progression and metastasis. Recent studies have provided insights into the roles of EVs in cancer stemness, tumor progression, TME, and formation of the metastatic niche. EVs from cancer cells contain unique proteins and nucleic acids that communicate with distant organs through body fluids. EVs can perform normal functions in the body and participate in different hallmarks of cancer progression, including TME and chemoresistance.^80^ Properly collected and isolated EVs can serve as biomarkers for cancer diagnosis in cases where traditional diagnostic methods are inaccurate.

## Authors contribution

Bhaumik Patel: conceptualization; Bhaumik Patel: writing – original draft; Bhaumik Patel and Sahdeo Prasad: writing – review & editing; Bhaumik Patel and Shreyas Gaikwad: visualization; and Sahdeo Prasad: supervision. All the authors critically revised and approved the final version of the manuscript.

## Ethics statement

None.

## Data availability statement

The datasets used in the current study are available from the corresponding author on reasonable request.

## Declaration of generative AI and AI-assisted technologies in the writing process

During the preparation of this article, AI or AI-assisted technologies has been only utilized to correct grammar and to simplify sentences. We, the authors, have reviewed and edited content and take full responsibility for providing publication-quality content.

## Funding

This research did not receive any specific grant from funding agencies in the public, commercial, or not-for-profit sectors.

## Conflict of interest

The authors declare that they have no known competing financial interests or personal relationships that could have appeared to influence the work reported in this paper.
